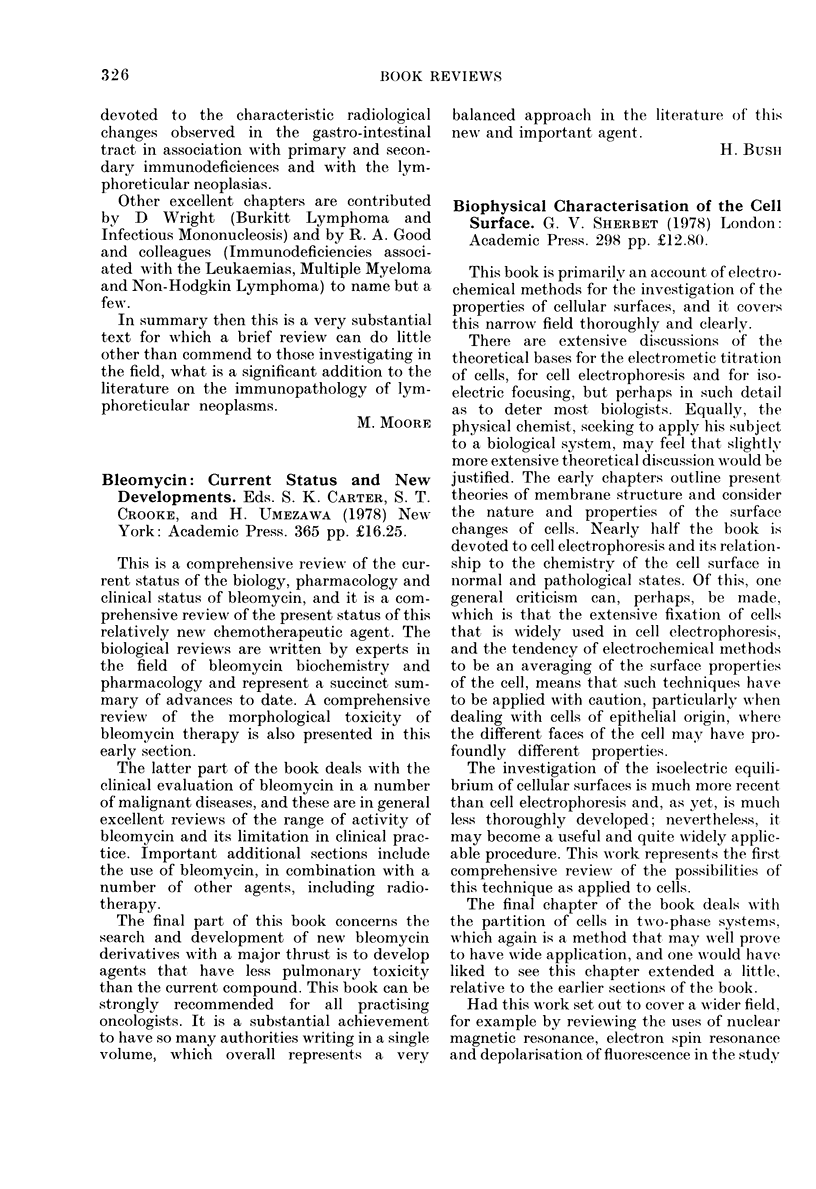# Bleomycin: Current Status and New Developments

**Published:** 1979-08

**Authors:** H. Bush


**Bleomycin: Current Status and New Developments.** Eds. S. K. CARTER, S. T. CROOKE, and H. UMEZAWA (1978) New York: Academic Press. 365 pp. £16.25.

This is a comprehensive review of the current status of the biology, pharmacology and clinical status of bleomycin, and it is a comprehensive review of the present status of this relatively new chemotherapeutic agent. The biological reviews are written by experts in the field of bleomycin biochemistry and pharmacology and represent a succinct summary of advances to date. A comprehensive review of the morphological toxicity of bleomycin therapy is also presented in this early section.

The latter part of the book deals with the clinical evaluation of bleomycin in a number of malignant diseases, and these are in general excellent reviews of the range of activity of bleomycin and its limitation in clinical practice. Important additional sections include the use of bleomycin, in combination with a number of other agents, including radiotherapy.

The final part of this book concerns the search and development of new bleomycin derivatives with a major thrust is to develop agents that have less pulmonary toxicity than the current compound. This book can be strongly recommended for all practising oncologists. It is a substantial achievement to have so many authorities writing in a single volume, which overall represents a very balanced approach in the literature of this new and important agent.

H. BUSH